# The Work of the Imperial Cancer Research Fund

**Published:** 1905-09-09

**Authors:** 


### HOSPITAL CLINICS.

### THE WORK OF THE IMPERIAL CANCER RESEARCH FUND.

(*continued from p.* 392).

PART II. of the recently-issued report of the above Fund deals with the growth of cancer (using the word generically as a synonym for malignant new growth) under natural and experimental conditions.

Regarding the processes which lead to the continued growth of transplanted malignant tumours when such transplantations are successful, there are two schools of thought. Dr. Bashford and his collaborators, on the basis of their transplantation experiments on mice, hold that the subsequent tumours are composed of the direct genealogical descendants of the cells artificially introduced. The majority of others who are committed to opinions assume the existence of some extraneous living agent or virus, whose presence in the cells calls forth a reaction comparable with that accompanying infective processes such as syphilis.

For two and a half years the officials of the Fund have attempted to propagate all the malignant growths they have received in a living and aseptic condition. In every case where the growths occurred in lower animals, portions of the growth were inoculated into animals of the same species, the growths so treated being derived from horses, dogs, cats, rats, mice, and carp. Almost every variety of malignant new growth was represented in the series; but, with the notable exception of mice, some 900 inoculations resulted in entirely negative results. In consequence, all the experimental successes with transplanted cancers have occurred in mice, and it is necessary to mention briefly the variety of malignant growth to which they are subject. Among 30,000 mice 12 instances of malignant disease appeared. The commonest is a tumour attacking the region of the mammæ, and histologically an adeno-carcinoma.

The success attending the transplantation of these tumours in mice was very variable. Some gave a uniformly low percentage of successes, while other specimens, histologically identical, and similar in their site of origin, yielded a much higher proportion of positive results. Further, all attempts to transplant growths into animals of a different species from those supplying the material have invariably failed. More than this, tumours which in one locality were readily transmissible to other animals of the same species in that locality, frequently aborted when introduced into similar animals of another district. Thus with a certain tumour, Professor Jensen of Copenhagen obtained during 23 generations a percentage of successful inoculations varying from 20 to 40. From this material 259 inoculations were made in this country, and resulted in success in $4\frac{1}{2}$ per cent. of the mice surviving the inoculation for a period of more than 14 days. Yet, when re-inoculations from these successes were performed upon English mice the percentage soon rose to the equal of that obtained by Jensen. It is clear, therefore, that minute racial distinctions play a part in determining susceptibility.

An important and copiously-illustrated section is devoted to an inquiry as to the source of the different constituents of artificially propagated new-growths. By the examination of successfully inoculated mice at varying periods after their inoculation the authors are able to state positively that in the case of propagated new-growths the parenchyma alone possesses the property of apparently continuous proliferation. The introduced stroma undergoes degeneration in the new host, and is replaced by supporting structures derived entirely from the tissues of the latter. This new stroma becomes ultimately identical in character with that of the original tumour, and thus the propagated tumour when fully established reproduces in every detail the characteristics of its original. This peculiar differentiation of the stroma of the new host into a type dictated by the tumour indicates that the reaction is a specific one for each variety of new growth. Such continuity of character can only be maintained by the influence of the elements which persist during artificial transplantation—namely, the parenchyma cells of the original growth. No information is forthcoming as to the mechanism of this process, but attention is drawn to the parallel thus furnished between the differentiation in the case of malignant disease and that displayed by the whole body in the course of its development. "The connective tissues and vascular structures of the different organs of the body are derived from elements of uniform character in the embryo. Their adult peculiarities are differentiations in accordance with the needs of the functional elements of the various organs." Similarly in the case of new growths it would appear that the character of the parenchyma dictates the character of the stroma supplied to it.

The constancy of this connective-tissue and vascular reaction for any one tumour indicates further that the cells of cancers, however little they appear to be differentiated in the direction of any particular type of tissue, yet possess permanent characters of their own strictly analogous to the higher differentiation of healthy structures. Just as the specific nature of the precipitin-reaction has revealed unsuspected differences between the body-fluids and tissues of even nearly related animals living on the same food, and as the propagation of tumours has revealed biological differences between animals of the same species but racially distinct, so the specific stroma-reactions of different tumours in animals of the same race reveal differences in cells histologically nearly related.

In order to determine the distinction between the mode of growth of cancer on the one hand and infective granulomata on the other, an exhaustive investigation was made of an infective venereal tumour occurring in dogs. This granuloma presents many points of similarity to a malignant growth, and has been described and argued upon as such. It is histologically similar to round-celled sarcoma, it grows partially from its own resources, confers no immunity upon survivors, and cannot be transmitted except to dogs. But it differs from new growths in the following particulars:—It is always directly transmitted: transformation of surrounding connective-tissue corpuscles into tumour-cells can be observed: the processes by which the tumours are formed though less in degree, are identical in character with those associated with the action of the tubercle or glanders bacillus: finally, the disease never occurs naturally in animals before sexual maturity, and is rare in old age. The non-malignant nature of this tumour deserves special recognition, since extensive experimental studies have been based upon it on the assumption of its malignancy; it is clear, therefore, that all inferences *ad hoc* are vitiated.

Metastasis-formation is rare in propagated cancer by comparison with the number of successful inoculations considered; but it occurs sometimes by the blood-stream, cancerous emboli plugging small arterioles, and supplying secondary foci of proliferation. Sometimes extension may be traced along lymph-channels, the masses of tumour-cells expanding the latter without destroying their lining-endothelium. The natural tendency of the growth is to progress by expansion: it is only when dense tissues oppose its march that infiltrative processes become evident. Under these latter circumstances extension takes place by means of narrow columns of cells, which gradually make their way through the less-resistant areas in their surroundings, but appear to exercise nothing like a digestive action in their neighbourhood.

In mice artificially inoculated with cancer, a specific cachexia does not accompany the new growth so long as no ulceration takes place. Delicate as are these animals, they are able to support large growths for months, both with and without metastases, and yet show no evidence of inconvenience. But as soon as rupture of the integument occurs the cancerous cachexia seen in man is very closely reproduced. It is therefore fairly certain that malignant new growths, *per se*, have no specific symptomatology.

(*To be continued.*)

